# The driving mechanism of large-scale comprehensive sports events on regional public sports activity growth: evidence from China's provincial panel data

**DOI:** 10.3389/fspor.2026.1883093

**Published:** 2026-08-10

**Authors:** Yu Wang, Wenjie Ma, Wei Zhao, Hongxing Chen

**Affiliations:** 1 Sports Department, Nanjing Institute of Technology, Nanjing, China; 2 Security Office, Nanjing Institute of Technology, Nanjing, China; 3 Institute of Sports Science, Nanjing Institute of Technology, Nanjing, China

**Keywords:** DID method, driving effect, large-scale comprehensive sports events, public sports activities, spacial layout

## Abstract

Based on the spatial difference-in-difference method, this study explores and analyzes the driving effect of large-scale comprehensive sports events on the growth of public sports activities in 31 provincial-level regions of China, from the perspectives of spatial aggregation, temporal trends, and regulatory mechanisms. The results indicate that during the 15-year period, the overall growth of public sports activities in various provinces demonstrated a spatial clustering trend (Moran's *I* = 0.21, *p* = 0.04), and the “high-high” clustering areas were highly correlated with the provinces hosting large-scale comprehensive events. Hosting large-scale comprehensive sports events can significantly increase the growth rate of regional public sports activities by approximately 26%, with a high growth period lasting for approximately 3 years, characterized by “large growth amplitude and short duration.” The spatial radiation range of large-scale comprehensive sports events is approximately 500 km, which has a significant driving effect on public sports activities within this distance. The competition achieves the growth of regional public sports services through three types of moderating factors: regional balance, policy environment, and factor growth. In a 15-year cycle, holding only one provincial-level large-scale comprehensive sports event yields the most significant driving effect on the growth of regional public sports activities, with a growth rate of approximately 10%. Adopting an interprovincial event hosting method of “two adjacent provinces” can generate more driving effects, with an average increase of approximately 9.2%. The hosting of large-scale comprehensive sports events should focus on building high-quality development policy concepts, optimizing regional sports resource balance, accelerating the transformation from a resource-driven to an innovation-driven one, and relying on the reasonable planning of event frequency and layout within and between provinces to achieve high-quality development of regional public sports services.

## 1 Introduction

Large-scale comprehensive sports events embody the comprehensive strength of a country or region, which can not only improve the level of regional competitive sports and economic development but also play a role in improving and driving the development of regional public sports activities (1, 2). The interactive development of China's large-scale comprehensive sports events and public sports activities has roughly progressed through three periods: The first is the foundation period. In 1959, the first National Games in Beijing became a milestone in the development of sports in New China. During this period, the main goal of large-scale comprehensive sports events was to embody the new spirit of the sports era, without yet achieving coordinated development with regional public sports activities. The second is the preliminary correlation period. After 1980, large-scale comprehensive sports events aimed to serve the national sports infrastructure, initially establishing linkage with public sports undertakings and promoting the enthusiasm of local residents wanting to participate in sports activities. The third is the period of comprehensive interaction. The large-scale comprehensive event system diversified and internationalized, forming a complementary pattern that included the Olympic Games, the Asian Games, and the National Games. The functions of these comprehensive games extended to many fields deeply related to public sports activities, such as the sports industry, the national fitness campaign, and the Healthy China initiative.

Large-scale comprehensive sports events achieve coordinated development of public sports activities by promoting the construction of sports facilities, stimulating sports service consumption, and increasing social access to venues (3–5). The results of collaborative simulation show that after increasing the purchase rate of sports services and the opening rate of sports facilities by 20%, the national fitness population demonstrated a steady growth trend (6). On the other hand, China's public sports activities are still beset with challenges such as insufficient special funding support, uneven distribution of infrastructure, and lack of professional technical guidance, which restrict the driving effect of large-scale events (7, 8). Therefore, hosting large-scale comprehensive games provides an opportunity to fully utilize policy resources during the preparatory period and improve the systemic shortcomings of public sports activities (9, 10).

This study explores the mechanisms through which large-scale comprehensive sports events drive the growth of regional public sports activities, focusing on the following four dimensions: First, we examine whether a substantial investment in large-scale comprehensive sports events can significantly promote the development of regional public sports activities. Such events directly contribute to the construction and transformation of regional public sports facilities, resulting in high-quality public sports and fitness facilities (11). Second, we analyze the spatial radiation effect of sports events and the adjustment mechanisms of distance factors. The social influence of high-level sports events is not limited to the host city but shows a strong positive spillover effect on the development of public sports activities at a certain spatial distance (12, 13). Third, we study whether public sports activities can maintain a benign and high-quality growth trajectory. The 2008 Beijing Olympics effectively promoted the development of mass sports and played a significant role in promoting individual outdoor activities and improving the exercise environment for residents. The 2024 Paris Olympics undertook frugal measures such as “vegetarian dining,” “rental tableware,” and “cardboard beds,” reflecting the deep motivation of large-scale comprehensive sports events to encourage a healthy lifestyle (14, 15). Fourth, we study the specific impact of event space layout on the growth rate of public sports activities. It is worth further exploring whether the layout of events within different provinces and between provinces has a significant impact on the growth rate of public sports activities in a region and what specific differences exist.

Based on the above-mentioned four dimensions, this paper selects provincial sports panel data from 31 provinces in China from 2008 to 2022 and applies the spatial difference-in-differences (SDID) method to quantitatively evaluate the driving mechanism of large-scale comprehensive sports events on the growth of regional public sports activities. The objectives, in particular, are as follows:

1. Verify whether large-scale comprehensive sports events can exert a significant pull on the growth of regional public sports activities and determine the time duration and spatial radiation characteristics of this process.
2. Examine the growth trajectory of public sports activities from the perspective of sustainable development and explore the demand for high-quality growth.
3. Describe the moderating role of spatial factors in the promotion of regional public sports activities from the perspectives of intraprovincial layout and interprovincial agglomeration and analyze efficient spatial factor–driven models.

## 2 Literature review

Large-scale comprehensive sports events represent the highest-specification and largest sports competitions held in China, which are closely linked to economic, social, and cultural development. Compared with general sports events, large-scale comprehensive sports events exhibit the following characteristics: First, such events obtain high-level policy support and have higher goals and functional positioning. This type of event enjoys special financial allocations and cross-departmental resource integration, with goals of boosting the economy, making breakthroughs in competitions, and developing sports. These aims not only provide athletes with international competitive platforms but also improve sports infrastructure and stimulate the enthusiasm of the masses to participate. For example, after the 2023 Hangzhou Asian Games, the utilization rate of national fitness facilities increased significantly, popularizing healthy lifestyles. The Winter Olympics also accelerated enthusiasm for technical exchanges and participation in ice and snow sports among residents in high-latitude areas (16, 17). Second, such events exert national and even global influence, driving the development of cross-regional economy and sports. Athletes in large-scale comprehensive events can reach thousands of people, and they need to invest a lot of money in infrastructure investment such as urban environment, event venues, and fitness roads, which strongly drive the development of sports in surrounding areas (18). After the 2008 Beijing Olympics, the number of people participating in physical exercise every week increased by 15%, and the usage of national fitness paths increased by 45%. The 2022 Beijing Winter Olympics proposed and achieved the goal of “encouraging 300 million people to participate in ice and snow sports” (19). Other important factors are the long preparation period and the significant influence of sports media. Taking the 2008 Beijing Olympics as an example, the preparation period was about 8 years. During this period, media publicity greatly increased the enthusiasm of the masses to participate in sports activities. The number of inquiries and memberships from related clubs doubled, driving a large number of people who had previously participated less in sports to join the national fitness movement.

Existing literature explores the driving mechanism of China's large-scale comprehensive sports events on the development of local public sports activities from multiple perspectives, including policy orientation, policy innovation, driving paths, and resource allocation. The policy research model based on the PMC index points out that the policy orientation of large-scale comprehensive sports events is conducive to promoting the development of the sports service industry, thereby increasing public participation in sports exercise (20). The policy orientation of sports events usually includes specific dimensions such as decision-making, market, and consumption. Measures centered on these dimensions often show significant positive promotion effects on the development of regional public sports activities (21). However, some regions face issues such as single policy effectiveness and insufficient incentive measures (22). Large-scale comprehensive sports events emphasize policy innovation, involving multiple aspects such as industrial restructuring and economic indicator–driven development in different host cities. Studies using propensity score matching-difference-in-differences show that policy innovation can rapidly promote regional economic development and exert a comprehensive and profound effect on social public services, with the impact process potentially lasting several years (23). In terms of driving mechanisms, large-scale sports events primarily rely on fiscal investment to promote the growth of public sports activities through upgrading sports facilities, increasing sports enterprises, stimulating sports consumption, and expanding the scale of the sports service industry (24). High-quality driving paths are a hot research topic, usually starting from consumption-driven factors such as participation in public sports activities, facility openness, and personnel allocation in sports social organizations, forming a virtuous cycle of event investment and social sports activities (25). Large-scale comprehensive sports events have wide-ranging influence. A qualitative study based on panel data from 15 provinces found that the diversified allocation model of large-scale comprehensive sports events was more conducive to enhancing the spatial aggregation and regional collaboration of sports resources, bringing about collaborative development of cross-provincial sports activities. This process shows a continuous upward trend in time series and significant heterogeneity in spatial distance (26, 27). In order to improve the efficiency of participation in public sports activities in China, geographical distribution patterns should be considered. Appropriate allocation models of policies, finances, sports resources, and social organizations should be established for different regions based on the Pareto optimality principle (28).

## 3 Theoretical mechanism

Reviewing the existing research, large-scale comprehensive sports events drive the growth of regional public sports activities primarily through the following paths:

1. The advantages of event policies contribute to the development of regional public sports activities. Building a fair and accessible public service system for national fitness is the cornerstone of building a sports power (29). Large-scale sports events are integrated into regional development plans and supported by national strategic policies during the planning and bidding stages, clarifying the layout of sports resources from the system and providing a prerequisite for regional residents to share public sports resources (30). During the preparation and competition stages, the event became a hot topic for promoting the national fitness strategy, and the continuous investment in sports facilities and fitness services promoted the “availability” and “accessibility” of regional sports resources (31, 32). As a supplement, comprehensive sports events represented by the Winter Olympics and the Winter Games also meet the diversified, professional, and personalized sports needs of China's high-latitude regions, improving people's experience of participating in sports and delivering high-quality sports services (33, 34).
2. The investment of event resources provides new growth points for the development of regional public sports activities. At present, the main reason for the significant disparities in the development of public mass sports in different provinces is fiscal expenditure, and insufficient funding leads to an imbalance or dysfunction in the allocation structure of public sports facilities and personnel in different regions (35, 36). Regional Finance has invested a lot of money in large-scale comprehensive sports events, creating new growth points for long-term support for limited public sports activities (31, 37, 38) and significantly improving the growth structure of a single public sports that relies on the financial support of mass sports. Research on the Asian Games shows that large-scale comprehensive sports events usually use basic urban renewal projects to achieve the reconstruction and transformation of living areas that lag behind other areas and promote modern social development, becoming a new growth point for promoting regional public sports activities (39). Therefore, whether it is financial support, resource rationing, or facility renewal, large-scale comprehensive sports events can introduce new growth points for regional public sports activities.
3. Events lead to the coordinated development of public sports activities through resource integration. The first point is investment resource coordination. Large-scale comprehensive sports events are usually jointly organized by multiple cities, and the resource integration investment model of “event + city” is formed through intraprovincial cooperation, which can achieve the rapid expansion of various resources in the short term (40, 41). Resource collaboration has further accelerated the interoperability of infrastructure and the sharing of sports resources, promoting the deep participation of citizens in the process of physical exercise (42). The second point is industrial consumption integration. Different from the unsustainable influence of individual events and ordinary events, large-scale comprehensive sports events involve the coordinated support of more sports organizations, which significantly drives the integration of the sports service industry and the level of mass sports consumption. The sports industry relies on events to generate consumer demand, bringing about the acceleration effect of public sports activities (3).

Based on the above analysis, hypothesis 1 is proposed: large-scale comprehensive sports events drive the growth of regional public sports activities through three types of models: policy advantages, resource investment, and resource integration.

The impact of large-scale comprehensive sports events on regional public sports activities is not comprehensive and balanced (43). If there is a lack of a long-term mechanism in the competition organization process, it will lead to difficulties in market-oriented operation in the postevent period, and it will be difficult to maintain the growth rate of the development of public sports activities (44, 45). On the other hand, the region that is more market-oriented is more likely to rely on economic foundation and service industry resources to achieve the long-term drive of events on public sports activities (44, 46, 47). Therefore, the heterogeneity of regional public sports activity growth driven by large-scale comprehensive sports events should be examined from two aspects: temporal heterogeneity and spatial heterogeneity.

1. The time node of event operation is heterogeneous in driving the growth of regional public sports activities. Large-scale sports events have similar operating cycles, and the phased differences in planning and design, production organization, and operation and maintenance management in the promotion process will bring about differences in the operational ecology of the event and the value-added time trend of the sports industry (48). In the later stage of the event, as policy dividends and resource advantages diminish, the growth rate of public sports activities will also differentiate over time (43). Other studies have shown that government support may be negatively correlated with the efficiency of regional public sports activities in the approach to large-scale events, reflecting excessive administrative intervention inhibiting market vitality and causing sports resources to crowd out (42). Therefore, the heterogeneity of time driven by large-scale comprehensive sports events to drive regional public sports activities should be analyzed from the aspects of event preparation and competition timing.
2. The spatial distribution of events also shows significant heterogeneity in the process of driving the growth of public sports activities. The effects of the efficiency and resource integration of public sports activities are characterized by spatial autocorrelation characteristics of “high-high” and “low-low” in geographical regions, and regions with high-dimensional clustering of sports innovation service enterprises are more likely to form a virtuous cycle of upgrading public sports services, achieving spillover growth of public sports activities (49, 50); On the other hand, excessive policy bias may absorb areas with sports resources that are too close to the event, hindering the development of mass sports (51, 52).

Based on further analysis, hypothesis 2 is drawn: the heterogeneity of large-scale comprehensive sports events on regional public sports activities is mainly manifested in two dimensions: time and space.

## 4 Research subjects and methods

### 4.1 Sample data

By referring to the classification method of “major sports events” (53), the study defines “large-scale comprehensive sports events” as competitive events that include multiple major sports events and have a fixed holding period; these events are important sports policies at the national or regional level. The sample treatment is as follows: Select provincial administrative regions for the sample. Excluding Taiwan Province and the Hong Kong and Macao Special Administrative Regions, panel data for 31 provincial-level regions were retained. In terms of time span, a total of 15 years from 2008 to 2022 was selected. Based on the competition and the year of hosting, a balanced panel data structure of 31 provinces over 15 years was finally formed (Table 1).

**Table 1:** National large-scale comprehensive sports events and host provinces from 2008 to 2022.

Holding time	Host city	Province	Event name	Preparation time	Event layout
2008	Qiqihar	Heilongjiang Province	National Winter Games	2006	Two cities in the province twice
2008	Beijing	Beijing	Beijing Olympics	2001	Two times in a single city
2009	Harbin	Heilongjiang Province	College Winter Games	2005	Two cities in the province twice
2009	Jinan	Shandong Province	National Games	2007	Single city
2010	Guangzhou	Guangdong Province	Guangzhou Asian Games	2004	Two cities in the province twice
2011	Shenzhen	Guangdong Province	World University Games	2007	Two cities in the province twice
2012	Changchun	Jilin Province	National Winter Games	2010	Single city
2013	Shenyang	Liaoning Province	National Games	2010	Single city
2013	Tianjin	Tianjin City	Tianjin East Asian Games	2011	Two times in a single city
2014	Nanjing	Jiangsu Province	Youth Olympic Games	2009	Single city
2016	Urumqi	Xinjiang Province	National Winter Games	2014	Single city
2017	Tianjin	Tianjin City	National Games	2016	Two times in a single city
2021	Xi'an	Shaanxi Province	National Games	2015	Single city
2022	Beijing	Beijing	Beijing Winter Olympics	2015	Two times in a single city

The dependent variable of the study is public sports activities, and the number of sports participants who exercise regularly in a region is often considered a key factor in measuring this indicator (36, 54). This study calculates the logarithmic growth rate in units of 10,000 people and obtains the value of this variable. The core explanatory variable is the hosting status of large-scale comprehensive sports events. If a provincial-level region hosts such events in a certain year, the subsequent years will take a value of 1, otherwise it is 0. Specific information on the settings, types, sources, definitions, etc. of various variables is given in Table 2.

**Table 2:** Main variable types and definitions.

Variable type	Variable name	Data source	Data definition
Explained variable	Growth of public sports activities before 2020	Sports Statistical Yearbook	The growth rate of public sports activities reflects the vitality of mass sports participation
	Growth data from 2020 to 2023	Provincial sports bureaus and sports guidance centers	Growth rate of indoor fitness personnel during the COVID-19 period
Explanatory variable	Holding large-scale comprehensive sports events	China Sports Yearbook and publicly available data from local governments	Variables for evaluating the policy influence of “large-scale comprehensive sports events”
Moderator variable	Regional equilibrium	Model calculation	The regulatory effect of balanced allocation of regional sports resources
	Policy innovation	Model calculation	The regulatory effect generated by policy environmental factors
	Element growth	Model calculation	The moderating effect of the growth of public participation factors in sports
Control variable	Collaborative variables that have impacts	Sports Statistical Yearbook	Variables that jointly participate in the influence in model (4)

### 4.2 Method

Large-scale comprehensive sports events may have spatial spillover effects on public sports activities, and the Moran *I* index is first used to test this effect (50). The formula for the Moran I index in global geographic space is as follows:

$$I\;=\;\frac{\sum_{i=1}^{n}\sum_{\;j=1}^{n}w_{ij}(x_{i}-\bar{x})(x_{j}-\bar{x})}{S^{2}\sum_{i=1}^{n}\sum_{\;j=1}^{n}w_{ij}}$$ (1)

In this formula, *n* represents the 31 provincial regions involved in the study, and *x_i_* and *x_j_* represent the spatial characteristics of province *i* and province *j*, respectively; $\bar{x}$ represents the mean spatial feature of provincial regions; *w_ij_* represents spatial weight; and *S^2^* represents variance. On this basis, the spatial clustering of specific provinces and the spatial spillover effects of individual or multiple regions are estimated through the local Moran *I* index. The calculation formula is as follows:

$$I_{i}=\;\frac{\left(x_{i}-\bar{x}\right)}{S^{2}}\overset{n}{\underset{\;j=1}{\sum }}A_{ij}(x_{i}-\bar{x})$$ (2)

Among them, *A_i_*_j_ represents the spatial adjacency weight matrix. When province *i* borders province *j* geographically, *A_ij _*= 1, otherwise, it is 0. Standardizing it can quantify the impact of neighboring provinces on the growth rate of public sports activities in each province and estimate spatial spillover effects based on the application of the time-varying DID model.

The basic model design based on spatial distance is as follows:

$$psag_{it}=\beta_{0}+\overset{3,000}{\underset{k=500}{\sum }}\varphi_{k}S_{it}^{k}+\beta_{1}\mathrm{DI}D_{it}+\lambda \mathrm{Control}s_{it}+\mu_{i}+\gamma_{t}+\varepsilon_{it}$$ (3)

Among them, the explained variable *psag_it_* represents the growth rate of public sports activities in each province over the past *t* years and measures the logarithmic growth percentage of the number of regular morning and evening exercises in each province. $S_{it}^{k}$ is a binary variable used to measure the influence of different spatial distance factors. *K* represents the spatial linear distance between the host cities of the province. Based on the spatial distance distribution of Chinese event host cities, the study selected 500 km as the measurement scale. If there is a province within [*k-*500, *k*] kilometers that has already hosted a large-scale event in year *t*, then the value of $S_{it}^{k}$ value is 1, otherwise it is 0. The coefficient vector *φ*_k_ reflects the spatial heterogeneity of the pulling process. If *k* is significantly positive at a certain value, it indicates that hosting large-scale comprehensive events at this spatial distance can significantly promote the growth of public sports activities in province *i*. When *φ*_k_ is set to 0, other items serve as the basis for the regression model. The value of DID_it_ in the year *t* and subsequent years is 1, otherwise, it is 0. If *β*_1_ is greater than 0 and significant, hypothesis 1 is verified; that is, large-scale comprehensive sports events will have a significant pulling effect on regional public sports activities. *μ*_i_ is the individual fixed effect, *γ_t_* is the time fixed effect, and *ε_it_* represents the standard residual term *i* = 1,2,…; 31 represents provincial regions, and *t* = 2008, 2009,…,2022 represents the year.

To avoid potential issues wherein baseline differential processing estimates do not conform to the stable unit treatment value assumption (50), a core spatial autocorrelation analysis SDID model was constructed on the basis of the above basic model:

$$psag_{it}=\beta_{0}+\rho Wpsag_{it}+\beta_{1}\mathrm{DI}D_{it}+\lambda \mathrm{Control}s_{it}+\mu_{i}+\gamma_{t}+\varepsilon_{it}$$ (4)

Among them, *W* is the preconstructed geographic distance spatial weight matrix, and ρ is the spatial autoregressive coefficient, which captures the spatial spillover effect of the explained variable between different regions. *Controls_it_* represents the control variables that have an effect on the growth of regional public sports activities; by referring to the provincial regional public sports activities evaluation index system (45), the control variables are set and defined as follows: (a) Mass sports expenditure. This provides the specific amount invested in mass sports to reflect the direct impact of capital investment on public sports activities; (b) Investment in sports venues. After including high-standard stadiums or ordinary public welfare grassroots venues built or maintained in the current year, if the coefficient is positive, the investment funds are tilted toward public sports activities; (c) The number of sports club organizations. This refers to the scale of social organizations that reflect mass sports activities; (d) Investment in domestic events. This includes local financial subsidies for different levels of sports events, reflecting the importance and organizational ability of the region to host sports competitions; (e) The number of sports fields built and maintained. This reflects the support capacity of regional public sports hardware facilities; (f) Investment in sports facility construction. This refers to the funds invested in the construction or upgradation of venues and facilities for sports competitions, training, and mass fitness activities. In terms of numerical processing, the variables of mass sports expenditure, investment in sports venues, and investment in domestic event indicators are taken as the logarithm of 10,000 yuan, and other indicators are taken as logarithms.

## 5 Results and analyses

### 5.1 Measurement results of spatial distance

**Figure 1:**
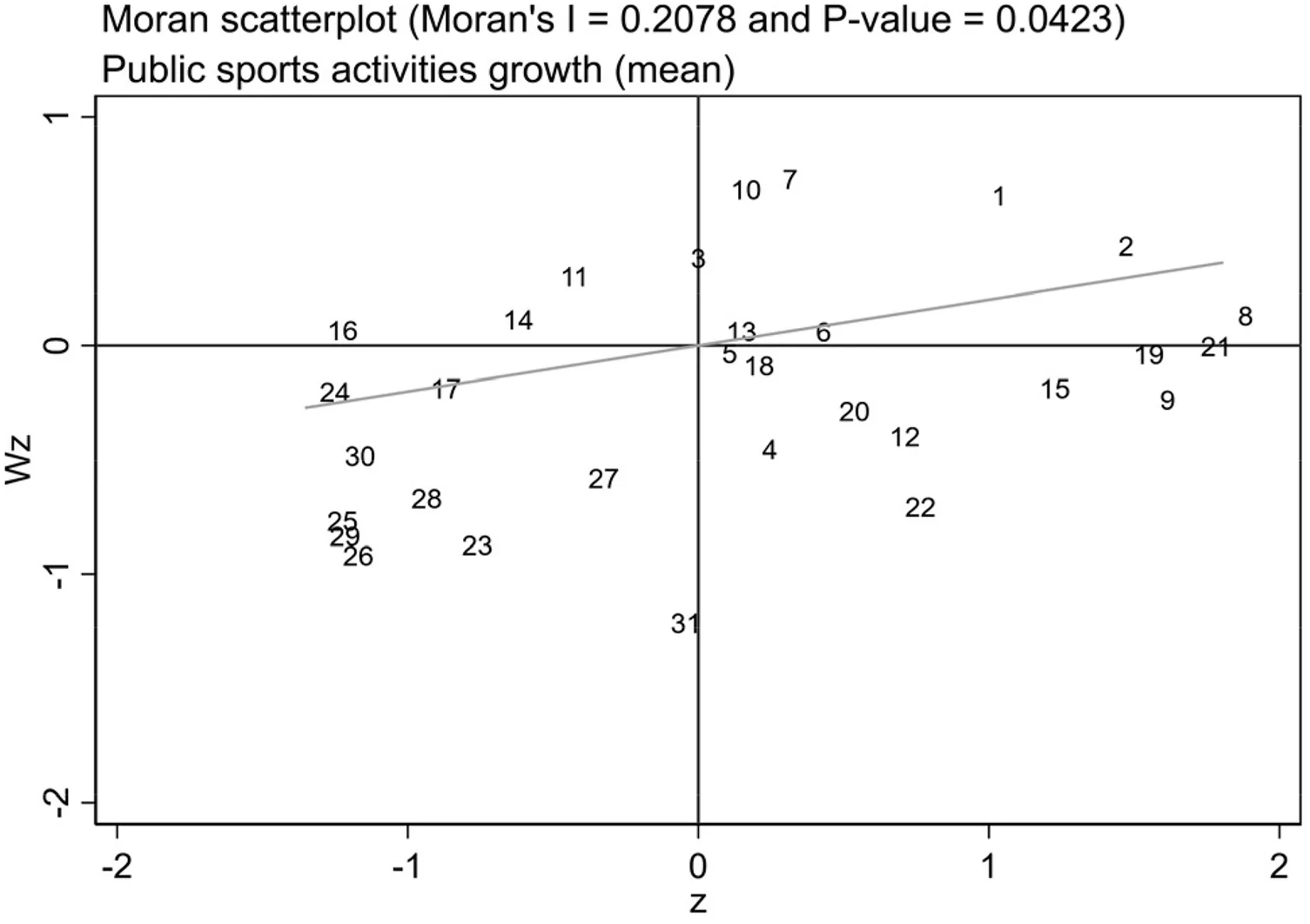
Local Moran I index for regional grouping (with province ID as the number).

This study focuses on the overall spatial clustering trend of various provincial regions over a period of 15 years. Therefore, based on the global formula (1), the arithmetic mean was applied to the regional values within each province to calculate the local Moran *I* index over this period. The Moran *I* index was found to be 0.2078 with a *p*-value of 0.04, reaching the level of spatial clustering significance (Figure 1). This indicates that the growth rate of public sports activities in various provincial-level regions exhibits a high-high clustering characteristic. Further based on formula (2), the local spatial Moran *I* index shows that the trend line is upward, and there are more provinces that have hosted events in the high–high clustering area, indicating that regions hosting large-scale comprehensive events have stronger spatial and geographical effects.

**Figure 2:**
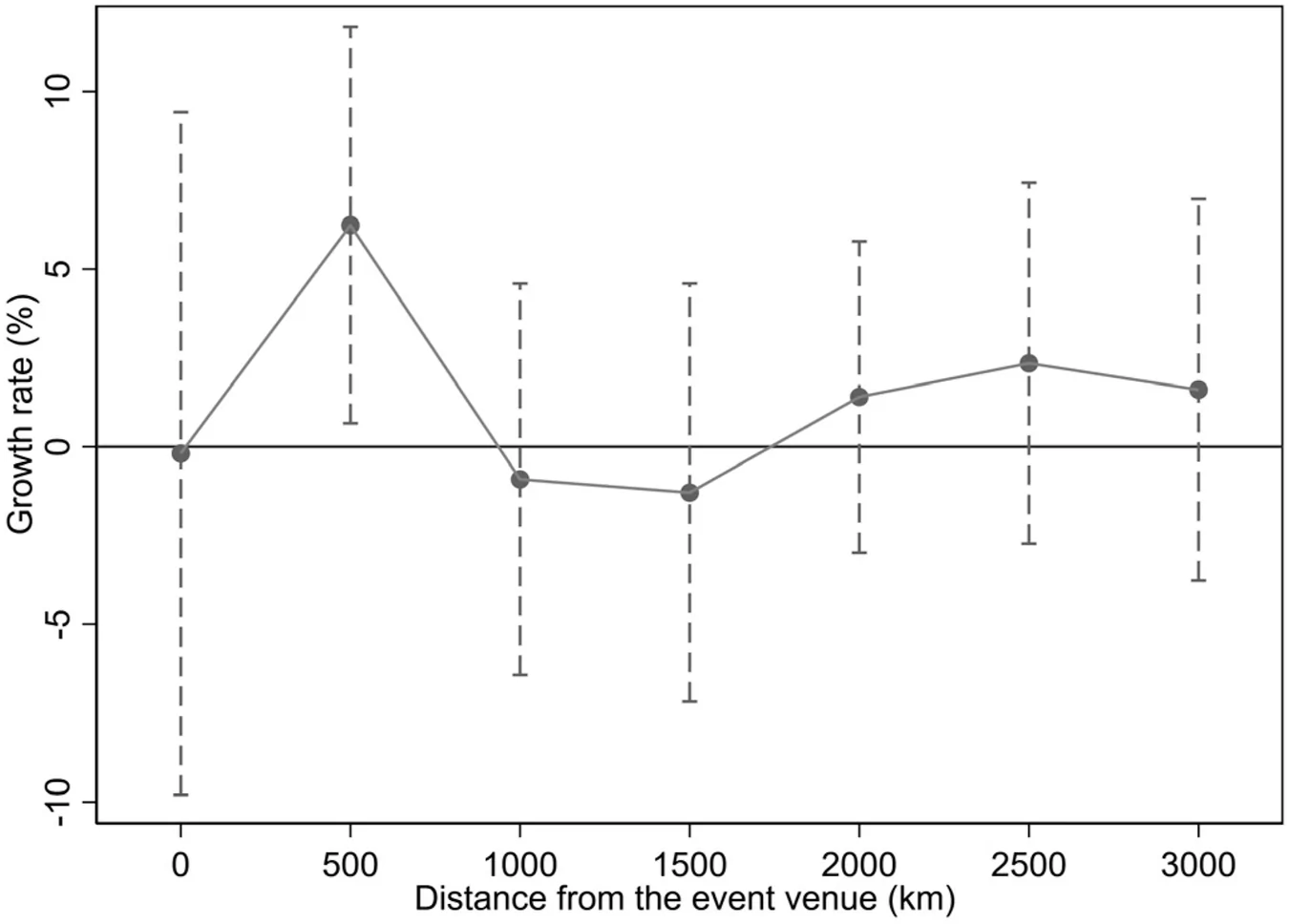
The impact of different distances on the growth of public sports activities.

The impact of large-scale comprehensive sports events on the growth of public sports activities at different distance scales is further determined, as shown in Figure 2.

Figure 2 shows the intuitive estimation results of separately controlling different spatial distances based on model (3). The overall performance of spatial heterogeneity is unstable. With the increase in the relative distance of the venue, holding large-scale sports events at a distance of 500 km will bring about a significant increase in public sports activities in the region, with a growth rate of approximately 6.24%. The other distance confidence intervals contained 0, and therefore, they did not reach a significant effect. The 0 point in the figure represents the distance between Beijing and Tianjin. The the adjacent situation in the municipalities directly under the Central Government with the closest spatial distance was examined, and the results showed no positive significant effect. This indicates that when the event locations are too close, large-scale comprehensive sports events may not directly drive the growth of public sports activities in these areas.

### 5.2 Basic regression

Based on a preliminary analysis of spatial distance factors, a correlation regression was established between large-scale comprehensive sports events and the growth rate of regional public sports activities. The results are presented in Table 3.

**Table 3:** The driving effect of sports events on the growth rate of public sports activities.

Variable	(1)	(2)	(3)	(4)
	Only control DID	Control all variables	Exclude provinces within 500 km of the events	Spatial autocorrelation model SDID
DID	9.0629*** (1.9091)	6.7996*** (1.8870)	7.2173*** (1.8276)	8.6745*** (2.4381)
Provincial effect	Control	Control	Control	Control
Annual effect	Control	Control	Control	Control
Rho				0.5808*** (0.0784)
*N*	465	465	341	465
*R*-squared	0.1849	0.2317	0.2741	0.0918

*, **, and *** represent the significance levels of 10%, 5%, and 1%, respectively, and the standard error is robust in parentheses.

In Table 3, column (1) shows the average driving effect of hosting large-scale comprehensive sports events on the growth rate of regional public sports activities, controlling only for double difference items. Column (2) displays the estimated results of adding all control variables on the basis of the first column. Column (3) shows that based on model (3), the estimated results of 23 provinces are retained after excluding provinces with provincial capitals 500 km away from the event venue in geographic space. The last column displays the estimated results based on model 4, excluding the influence of spatial radiation factors. The results indicate that, considering only the direct driving effect, hosting large-scale comprehensive sports events can also play an important role in driving the growth of public sports activities in the province, with an average growth rate of approximately 6.8 percentage points, indicating a significant increase. After excluding spatial radiation factors, this growth rate significantly increased to 7.22–8.67 percentage points. The SDID model shows that the spatial correlation coefficient ρ=0.58 is significantly positive. It can be considered that the real local direct effect of this type of event on the growth of public sports activities is approximately 8.67%, and the driving effect is very significant.

### 5.3 Robustness test

**Figure 3:**
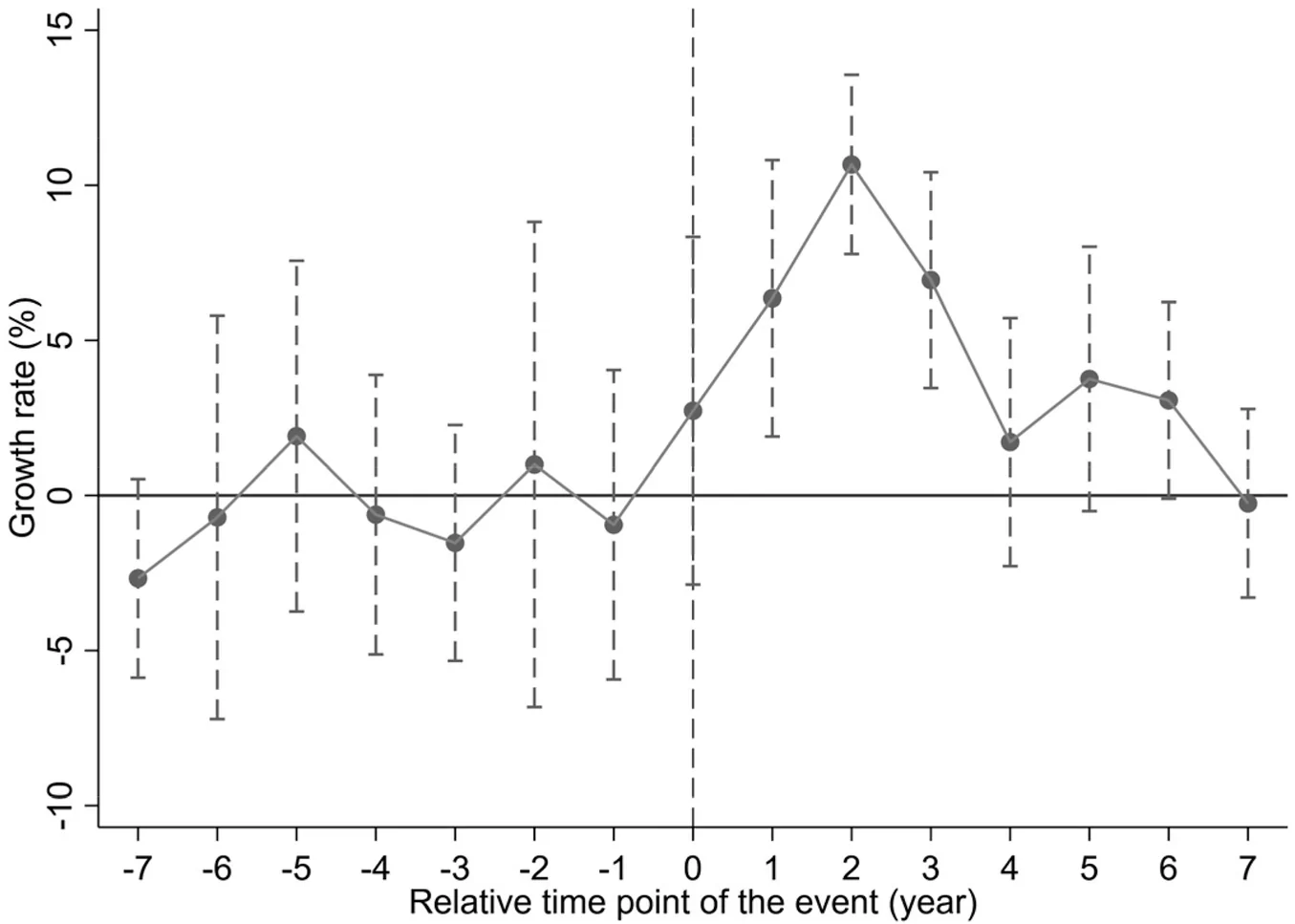
The heterogeneity of the timing of events on the growth of public sports activities.

**Figure 4:**
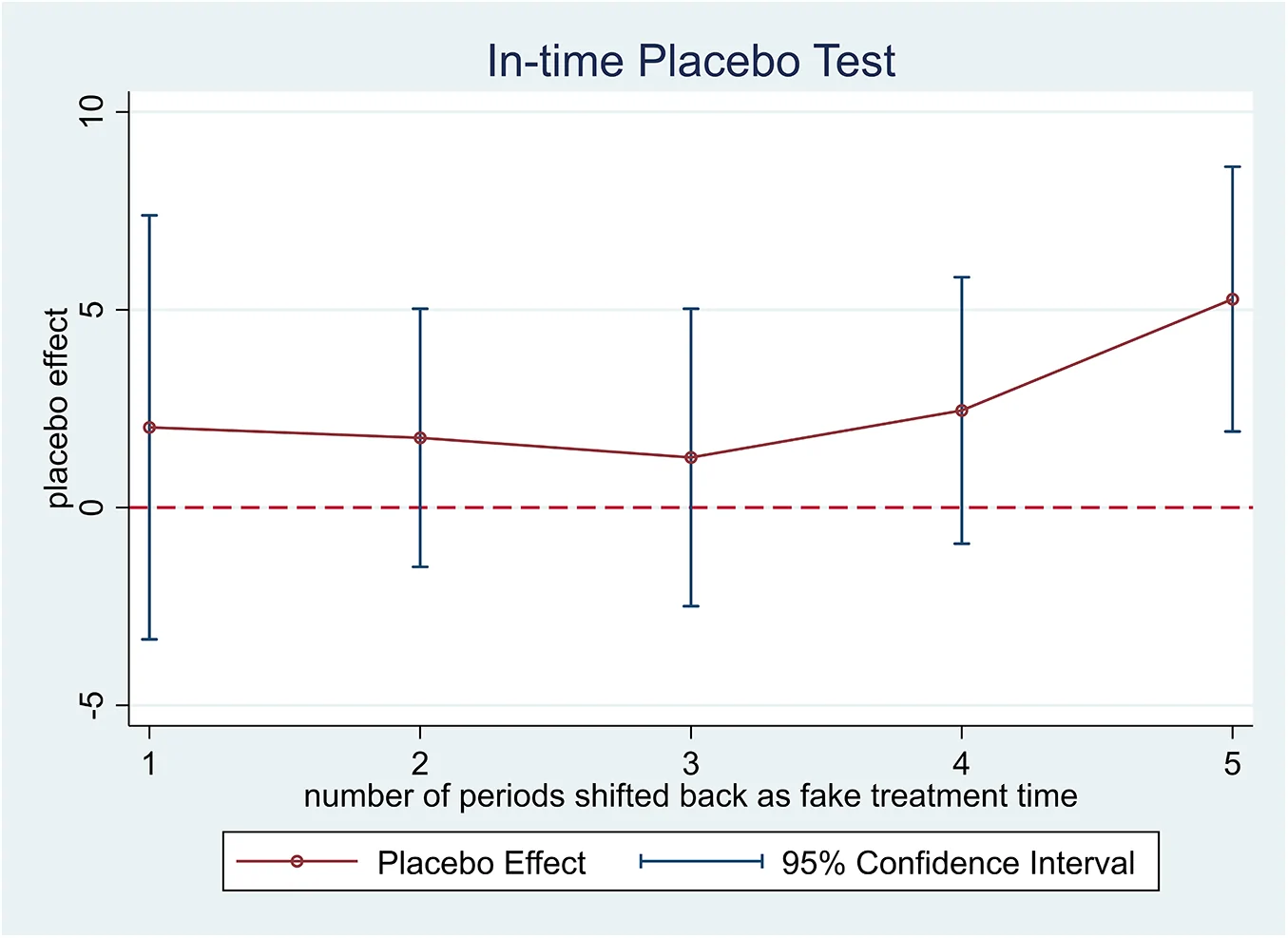
A time placebo test with a five-period delay.

**Figure 5:**
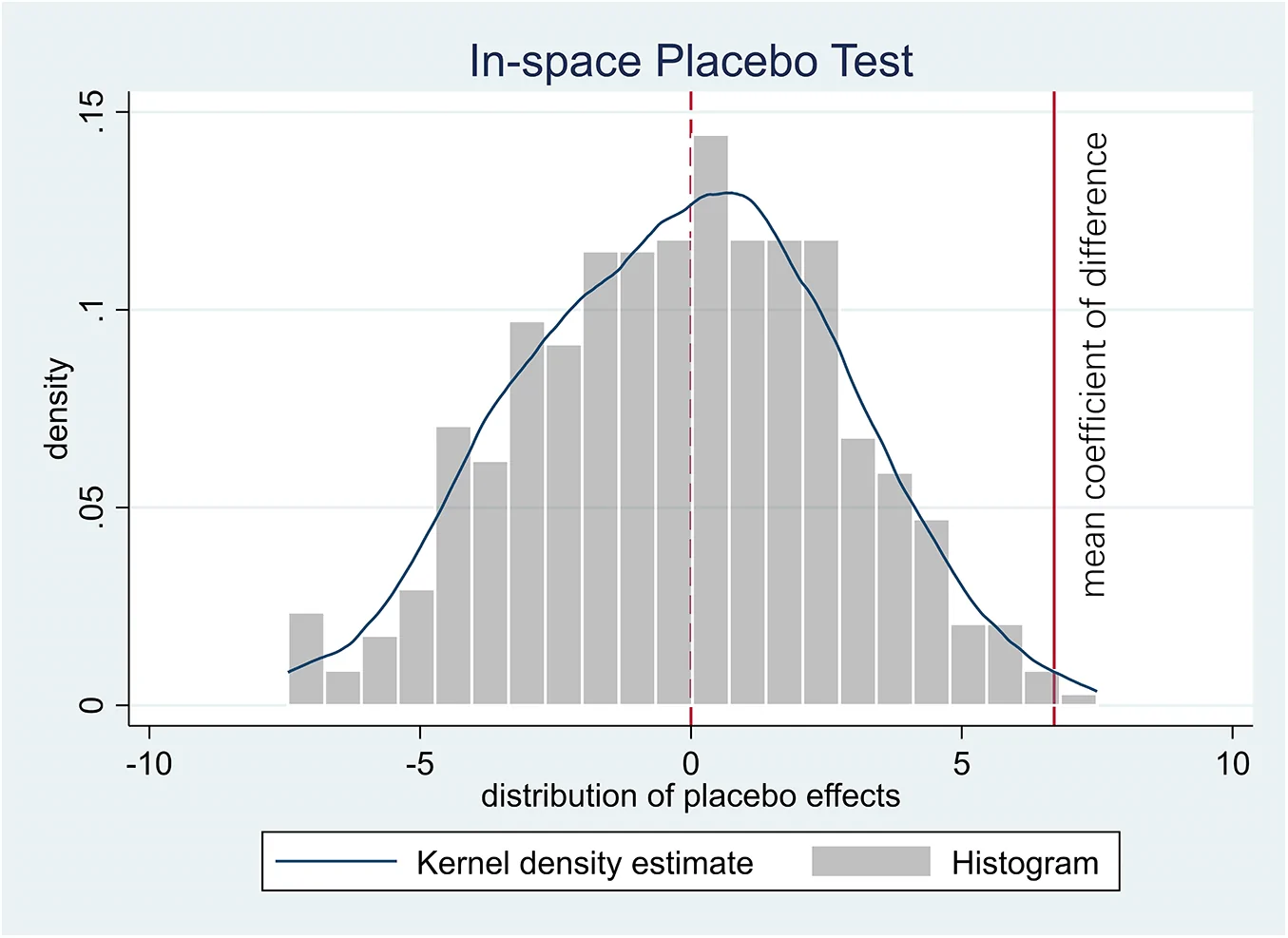
A spatial placebo test (random sampling 500 times).

1. Parallel trend test. First, a parallel trend test was conducted on the timing of the event hosting policy, and the results are shown in Figure 3.
  According to Figure 3, taking the 8th year before the event as the base period, in the 7 years before the “large-scale comprehensive sports event” was held, the relative difference in the driving level of regional public sports activity growth between the experimental group and the control group provinces included 0 in the 95% confidence interval, indicating no significant difference. From the moment of the competition, the pulling speed suddenly increased significantly and reached a positive and significant level in the first year, becoming a relatively stable turning point with a duration of approximately 3 years. Because of the lag effect of event policies, the growth of public sports activities in the region reached its peak in the second year, with a value exceeding 10%. In the fourth year after the event, the pulling effect tended to stabilize and was no longer significant. Based on the results in Table 3, it can be concluded that large-scale comprehensive sports events will bring a significant acceleration effect at an average of 8.67% to the growth of local public sports activities for 3 consecutive years, with an overall increase of 26%.
2. Placebo test: According to the presupposition, once a large-scale comprehensive sports event is held, it is regarded as a continuous processing of the policy, and the placebo test is implemented considering two aspects: time and space. First is the time placebo test: The observations were removed after the event time, the data of the event time lag of five periods were set as a pseudoprocessing period under the condition that the data are converted to the non-balanced panel format, and then a double differential examination of the significance of placebo was conducted. The standard error results of robust clustering showed a positive trend, and the confidence intervals of 1–4 periods included 0, indicating that the placebo effect was not significant within 4 periods of the event time (Figure 4). Second is the spatial placebo test: Without changing the time point of the event, the “held” and “non-held” states of each province and region were randomly replaced, a new placebo sample for double difference estimation was formed, and this process was repeated 500 times to estimate the *p*-value. The results showed that the placebo effect was located on the left side of the mean coefficient of difference, with a two-sided *p*-value of 0.018 and a right-sided *p*-value of 0.006, which were significant at the 5% and 1% levels, respectively, indicating a significant treatment effect (Figure 5).
3. Impact of other sports events. Each province usually holds different types of sports events, and whether these events will have an impact on the growth of public sports activities in the region is a factor that cannot be ignored. Based on the feature classification of sports events (55), this study selected three types of sports competitions. (a) Professional leagues and tournaments. Games such as the Chinese Super League, Modern Pentathlon Championship, and CBA All Star Game may be held at any time; (b) Specialized events such as the F1 Chinese Grand Prix, Tennis Open, and Race Walking Championships; (c) International regular events held in China, such as the Norske Grand Prix and Asian Beach Games. In order to estimate the marginal impact of these events, control for three types of sports events was added on the basis of model (4), labeled as D1*_it_*, D2*_it_*, and D3*_it_*, respectively. If province *i* holds professional leagues and championships in year *t*, then in year *t* and subsequent years, the D1 variable of region *i* is assigned a value of 1, and all other variables are assigned a value of 0; D2 and D3 also adopt the same scheme. The estimated results are presented in Table 4.
  The results indicate that after the introduction of regular sports events, the driving effect of large-scale comprehensive sports events on regional public sports activities is still significantly positive. The standard deviation values of the mixed effects of the three types of events are relatively large and have not reached a significant level. Therefore, after this round of testing, the impact of other common types of sports events on regional public sports activities can be excluded, and the unique significance of large-scale comprehensive sports events as a driving effect can be determined.
4. Impact of sample structure event timing and COVID-19. The specific processing results are presented in Table 5.

**Table 4:** Impact assessment of other sports events.

Variable	(1)	(2)	(3)	(4)
	Professional leagues and tournaments	Special events	General international competitions	Mixed effects
DID	8.8712*** (2.4286)	9.2071*** (2.4505)	9.0174*** (2.4568)	7.8467*** (2.4557)
D1	2.9792 (2.3827)			−1.1492 (2.2546)
D2		3.2852 (2.1029)		0.1998 (2.0502)
D3			5.3377 (3.3441)	4.2279 (3.1061)
Provincial effect	Fixed	Fixed	Fixed	Fixed
Annual effect	Fixed	Fixed	Fixed	Fixed
Rho	0.5880*** (0.0739)	0.5830*** (0.0761)	0.5729*** (0.0808)	−0.7414*** (0.1116)
*N*	465	465	465	465
*R*-squared	0.0980	0.099	0.0976	0.0322

*** represents the significance level of 1%, and the standard error is robust in parentheses.

**Table 5:** Influence of sample structure, event time, and COVID-19 epidemic.

Variable	(1)	(1)	(3)	(4)
	Retracting	Lag effect	Excluding earlier impact	COVID-19 impact
DID	7.1702*** (2.2929)	8.3894*** (2.5969)	9.6024*** (2.798)	0.8845*** (0.0768)
Provincial effect	Fixed	Fixed	Fixed	Fixed
Annual effect	Fixed	Fixed	Fixed	Fixed
Regional effects	Nope	Nope	Nope	Fixed
Rho	0.5927*** (0.0744)	0.5917*** (0.0756)	.5845397*** (0.0783)	−0.5555*** (0.1361)
*N*	465	465	465	465
*R*-squared	0.0880	0.2449 (0.0864)	0.2498 (0.0878)	0.2968

*** represents the significance level of 1%, and the standard error is robust in parentheses.

Among them, column (1) is the estimation result after removing potential anomalies and retracting 5%; column (2) considers the lag effect of resource allocation of sports events, replaces the processing time of the event time with the event preparation time node, and evaluates the pulling effect on regional public sports activities; column (3) reports the results of excluding the earlier impact, which is to remove the provinces that held events before the two competition cycles, that is, exclude the regions that held large-scale comprehensive sports events after 2000, and evaluate the growth effect of events and public sports activities. The first three columns of the results indicate that large-scale comprehensive sports events have shown a significant promoting trend on the growth of public sports activities in the region, with a positive and significant spatial autoregressive coefficient ρ close to 0.6, indicating that the impact process shows strong positive radiation in the geographical area.

The fourth column separately lists the impact of COVID-19 factors. Since the occurrence of COVID-19 in China at the beginning of 2020, outdoor public sports activities decreased significantly, and therefore, this study chose the growth rate of indoor sports participation as a proxy variable to build a spatial analysis model. By referring to the research plan of Yin et al. (56), the interaction term TDID * COVID-19 between double difference variables and epidemic impact factors was constructed for analysis. The regression results show that the coefficient of the core variable is 0.88 and significantly positive at the 1% statistical level, with a corresponding *z*-value of up to 11.52, indicating that the opening of indoor sports venues in the region has led to an extremely significant positive promotion of residents’ participation in physical exercise. The spatial autoregressive coefficient ρ=−0.56 is very significant, indicating that there is a significant negative spatial siphon effect within the research sample interval; that is, the expansion of indoor sports resources in neighboring areas will divert the local sports exercise population, partially offsetting the policy dividends of local indoor sports construction. In summary, in the 3 years after 2020, during COVID-19, the impact of large-scale sports events on residents’ exercise behavior is not a homogeneous positive diffusion in the spatial dimension but a heterogeneous feature of “core agglomeration and peripheral diversion.”

### 5.4 Moderating mechanism

#### 5.4.1 Moderating effect of regional equilibrium

Large-scale comprehensive sports events have brought new growth points to regional public sports activities, and their growth rates show a parallel trend of faster growth rates as they approach the event time (Figure 3). It is not difficult to infer that treating public sports activities in provincial-level areas as a cross-sectional individual may have a faster pulling effect in less developed regions when jointly influenced by event timing policies. Therefore, it is necessary to explore the moderating effects of public sports activities in regions with different levels of development during the competition period. The method is to introduce a new variable *psagini_it_* based on the core space regression model (4) to reflect the initial growth rate of public sports activities in the region before hosting large-scale comprehensive sports events. According to Figure 4, the initial time is determined to be 4 years before the event begins, and the estimated results are presented in Table 6.

**Table 6:** Moderating effect of regional equilibrium.

Variable	(1)	(2)	(3)
	Growth rate during 2004–2008	Growth rate during 2004–2006	Initial growth rate in 2004
DID	6.180202*** (2.4408)	5.7964** (2.4766)	6.4681*** (2.4249)
DID*psagini	−0.6237^**^ (0.2979)	−0.8979*** ( 0.4234)	−0.8923* (0.4921)
DID + interaction term			
*psagini* 1/4th quantile	9.8546*** (2.7083)	8.6359*** (1.4789)	7.0037*** (1.3345)
*psagini* 1/2 quantile	5.0201*** (1.1852)	5.6676*** (1.3621)	4.9575** (1.8364)
*psagini* 3/4 quartile	−3.8072* (1.7583)	−2.2551 (2.4088)	−3.6934 (4.9824)
Provincial effect	Fixed	Fixed	Fixed
Annual effect	Fixed	Fixed	Fixed
Rho	−0.6799*** (0.1168)	−0.6842*** (0.1159)	−0.6873*** (0.1162)
*N*	465	465	465
*R*-squared	0.0792	0.0784	0.0764

*, **, and *** represent the significance levels of 10%, 5%, and 1%, respectively, and the standard error is robust in parentheses.

The regression results in column (1) of Table 6 show the growth rates of public sports activities in various provinces and regions from 2004 to 2008. The other two columns show the regression results of the growth rate of public sports activities from 2004 to 2006 and the initial growth rate in 2004. The results showed that the double difference item was always positive and significant, the interaction term was always negative, and the initial growth rate of public sports activities showed a downward trend with the increase of quantiles, indicating that the driving effect of large-scale comprehensive sports events on the growth of public sports activities in the region is weakening. Overall, the level of regional public sports activities is driven by large-scale comprehensive sports events, with a more significant driving effect on underdeveloped areas.

#### 5.4.2 Moderating effect of the policy environment

Large-scale comprehensive sports events have brought a favorable public sports policy environment to the region, which can make up for the shortcomings of relevant policies and promote the rapid development of regional public sports activities. Based on this viewpoint, the hosting of sports events should bring greater impetus to regions with weaker initial sports policy environments. According to model (4), this study introduces the policy moderating variable *policyini* to reflect the policy environment associated with major sports events in the region and estimate the relevant policy moderating effects. The DEA model is a commonly used tool for evaluating policy implementation, which constructs the production frontier through linear programming and quantifies the efficiency of input-output of the evaluation object. By referring to the indicator design of the DEA evaluation model for sports policies (54), this study determines the input indicators as the annual growth rate of competitive sports expenditure and the annual growth rate of sports training expenditure. The effectiveness output indicators selected two indicators: the number of registered social sports instructors/10,000 people and the area of sports venues/10,000 people, respectively, reflecting the support potential of regional competitive sports and mass sports. After normalizing the data, the DEA efficiency values of two policy inputs in district *i* in year *t* are estimated and summed to determine the impact of the policy environment on public sports activities. The estimated results are presented in Table 7.

**Table 7:** Moderating effect of the policy environment.

Variable	(1)	(2)
	Policy environment effect	Effect with control variables
DID	6.734*** (2.4045)	6.3169*** (2.4430)
DID*policyini	−0.9937* (0.6201)	−1.1059* (0. 6201)
DID + interaction term		
*Policyini* 1/4 quartile	9.4414*** (1.4183)	7.1676*** (1.3073)
*Policyini* 1/2 quantile	7.7156*** (1.8438)	5.0147** (1.8041)
*Policyini* 3/4 quartile	7.6458*** (1.8651)	4.9277* (1.8299)
Provincial effect	Fixed	Fixed
Annual effect	Fixed	Fixed
Rho	−0.7402*** (0.1119)	−0.6871*** (0.1163)
*N*	465	465
*R*-squared	0.0313	0.0761

*, **, and *** represent the significance levels of 10%, 5%, and 1%, respectively, and the standard error is robust in parentheses.

Table 7 shows the regression results of policy environment factors with and without control variables. The results showed that the coefficient of interaction terms was negative and reached a significance level of 0.01, indicating that the original sports policy environment in the region has a moderating effect on the growth of public sports activities driven by large-scale events. Because of the significant positive effect of the double difference coefficient, the negative moderating effect is stronger for policy advantageous regions. Column (2) shows that in regions with policy evaluation scores in the first quartile, hosting large-scale comprehensive sports events will lead to a 7.17% growth rate in public sports activities, which is very significant compared with the third quartile regions (4.93% growth rate) (*p* = 0.000). From another perspective, in areas with relatively backward policy environments, hosting large-scale comprehensive sports events will bring greater growth to public sports activities.

#### 5.4.3 Moderating the effect of element growth

The growth of regional public sports activities driven by events generally shows the characteristics of rapid growth and short duration in terms of time trend. Whether this growth process meets the requirements of high-quality development is the focus of research and needs to be further analyzed. By referring to the definition of the efficiency driving indicators of sports public services (45, 57), this study splits the growth rate of public sports activities into two parts: investment-driven and consumption-driven. Among these, the investment driven growth rate includes two specific indicators: administrative investment and labor expenditure, which mainly involve the financial allocation and additional human factors of public sports investment in province *i* in the year *t*. The consumption-driven growth rate also includes two indicators: mass fitness and sports industry, reflecting the scope of industrial consumption driven by public sports activities in the province during the same period. Taking the logarithm of each indicator in 10,000 yuan and calculating the growth rate, the driving mechanism of regional public sports activity growth is analyzed, and the results are presented in Table 8.

**Table 8:** Moderating effect of element growth.

Variable	Investment-driven		Consumption-driven	
	Administrative input	Labor expenditure	Mass fitness	Sports industry
DID	7.0282*** (2.5264)	4.1261 (2.8897)	−1.1723 (2.4697)	1.9444 (9.2328)
Provincial effect	Fixed	Fixed	Fixed	Fixed
Annual effect	Fixed	Fixed	Fixed	Fixed
Rho	0.3345* (0.1833)	0.09233 (0.3547)	0.2479* (0.1443)	−0.8047*** (0.1078)
*N*	465	465	465	465
*R*-squared	0.0369	0.0360	0.0568	0.0148

* and *** represent the significance levels of 10% and 1%, respectively, and the standard error is robust in parentheses.

Table 8 shows that the DID coefficient values maintain a positive and significant investment drive for the growth of regional public sports activities. Among them, the administrative input variable coefficient is 7.03, which indicates a very significant level of 0.01, and the labor expenditure coefficient is 4.13, which is not a desired level of significance. The factors related to consumer driven mass fitness and sports industry have not reached a significant level, failing to promote the growth of public sports activities. Therefore, the current growth momentum of regional public sports activities is mainly achieved through investment channels. Due to insufficient sports consumption, it is difficult to form a virtuous cycle of industrialization, and the significant growth of public sports activities can only be maintained for a relatively short period of time.

### 5.5 Influence of events layout

#### 5.5.1 Layout of events in the province

**Figure 6:**
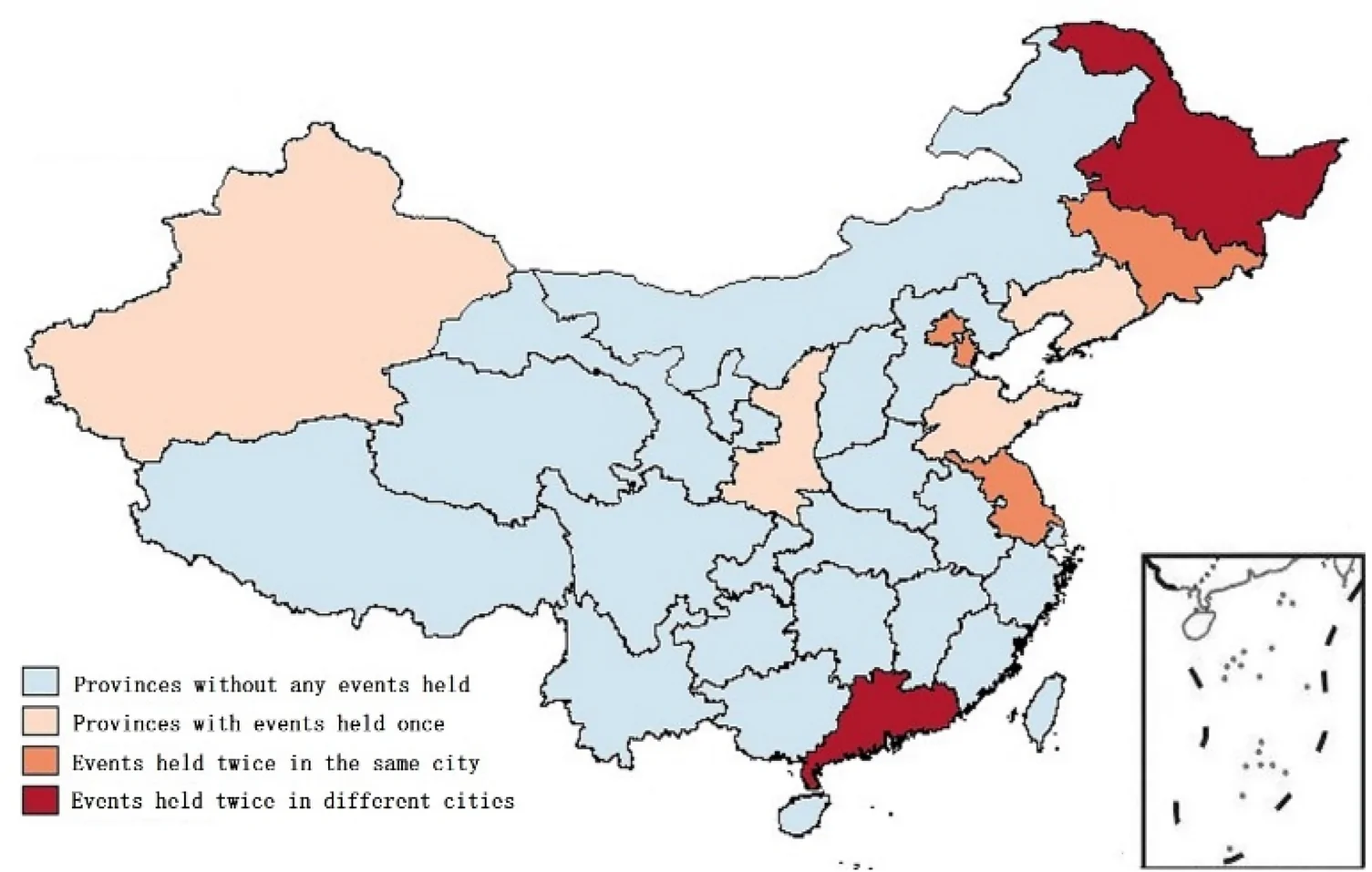
A layout of provincial events.

During the 15-year research period, provinces and regions hosting large-scale comprehensive sports events exhibited different frequencies and distribution characteristics (Table 1), with three layouts displayed within the province (Figure 6). The existing spatial analysis models cannot effectively handle these differences. Therefore, based on the double difference item in model (4), this study introduces the virtual variable *layout_p_* of the event layout, constructs a DID * *layout_p_* triple difference item, and regresses the growth of regional public sports activities to distinguish the driving efficiency differences of the three provincial event layouts. The estimated results are given in Table 9.

**Table 9:** Spatial layout and growth of public sports activities in the province.

Variable	(1)	(2)	(3)
	Province with events held once	Events held twice in the same city	Events held twice in different cities
DID*layout*_p_*	9.9496*** (3.0138)	7.6311* (4.007)	−8.0893 (7.6195)
Provincial effect	Fixed	Fixed	Fixed
Annual effect	Fixed	Fixed	Fixed
Rho	0.5956*** (0.0743)	−0.7011*** (0.1146)	−0.7171*** (0.1107)
*N*	465	465	465
*R*-squared	0.0872	0.0591	0.0522

*, and *** represent the significance levels of 10% and 1%, respectively, and the standard error is robust in parentheses.

Table 8 shows that the layout of the three types of events will have a significant impact on the growth of regional public sports activities. Provincial regions that only host such events once have a more significant driving effect on the growth of their public sports activities, with an average increase of 9.95% and reaching a very significant level. The spatial effect coefficient ρ is 0.6 and significant, indicating the generation of positive radiation effects. Increasing the frequency of events (such as 2 within 15 years) may have a restraining effect on the growth of public sports activities. Among them, holding two events in the same city can still maintain significant positive growth, but the growth rate drops to 7.63%; Holding two events in different cities will have a strong negative effect, reducing the growth rate of public sports activities by approximately 8.1%. Therefore, taking a 15-year cycle as an example, holding only one large-scale comprehensive sports event is the most effective way to promote the growth of public sports activities in the region.

#### 5.5.2 Layout of interprovincial events

**Figure 7:**
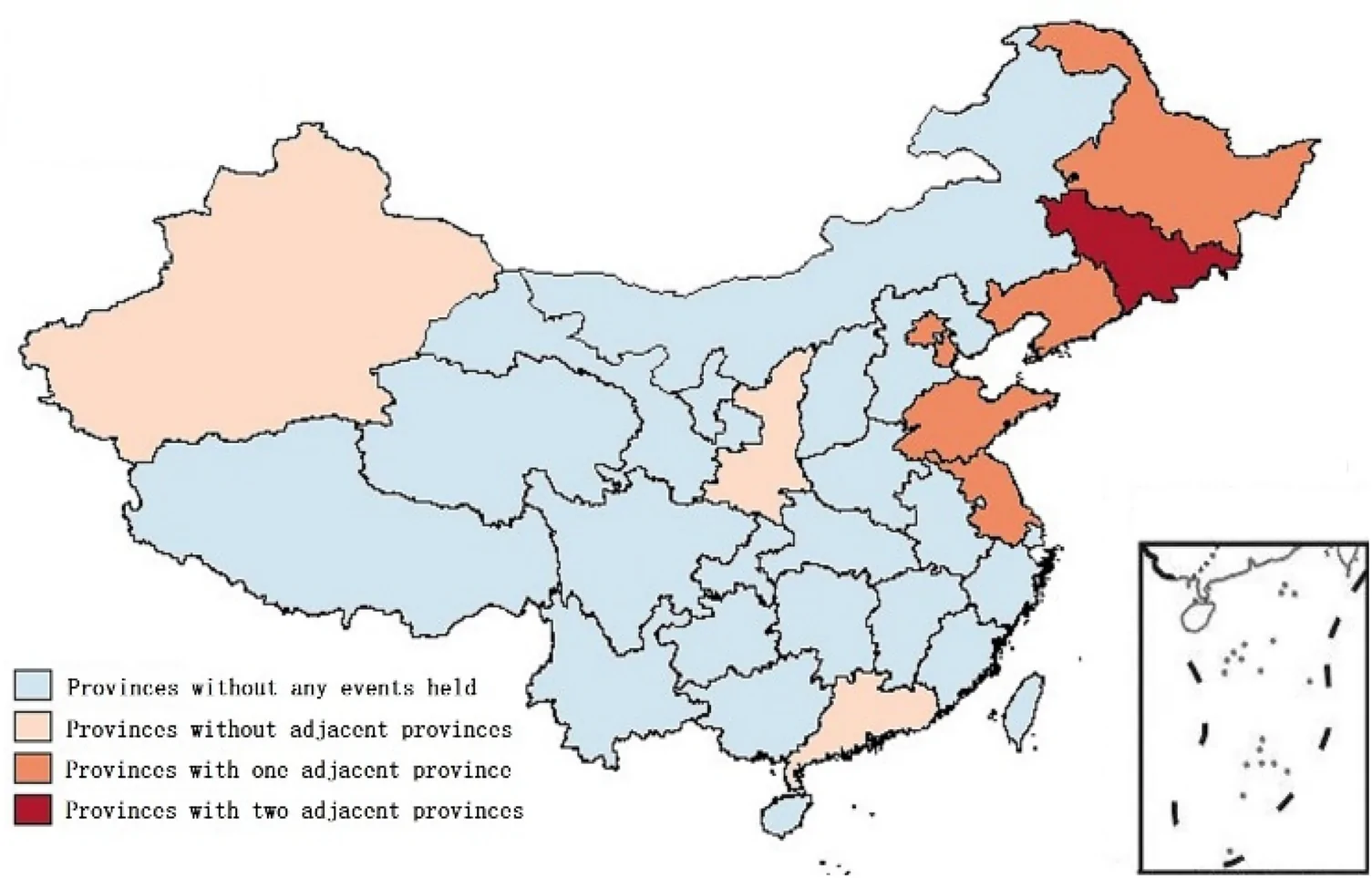
A layout of interprovincial events.

Based on the analysis of different spatial distances (Figure 2), large-scale comprehensive sports events will have a significant positive driving effect on regional public sports activities at a distance of 500 km (Figure 2). This radiation distance may exceed most provincial regions and reach neighboring provinces, and therefore, further exploration of this issue is needed from the spatial layout of interprovincial events. During the sample study period, each region also exhibited three characteristics in terms of provincial distribution: spatial isolation, one neighboring province hosting similar sports events, and two neighboring provinces hosting events (Figure 7). Therefore, the study introduced the dummy variable layout_i*p*_ and constructed a DID * layout_i*p*_ triple difference item to estimate the driving effect of three interprovincial spatial event layouts on the growth of regional public sports activities. The results are presented in Table 10.

**Table 10:** Interprovincial spatial layout and growth of public sports activities.

Variable	(1)	(2)	(3)
	Province without adjacency	Province with one adjacency	Province with two adjacencies
DID*layout_i*p*_	6.658* (4.0342)	9.1638** (3.7690)	4.9917 (5.6905)
Provincial effect	Fixed	Fixed	Fixed
Annual effect	Fixed	Fixed	Fixed
Rho	0.5967*** (0.0751)	0.6005*** (0.0743)	−0.7158*** (0.1120)
*N*	465	465	465
*R*-squared	0.0692	0.0736	0.0524

*, **, and *** represent the significance levels of 10%, 5%, and 1%, respectively, and the standard error is robust in parentheses.

Table 10 shows that regional public sports activities exhibit significant positive growth when there no or only one neighboring province hosts large-scale comprehensive sports events. Among them, without adjacent provinces, the growth rate of public sports activities is 6.66%, while with one adjacent province, the growth rate is faster, reaching 9.16%. There are two adjacent provinces whose growth rate does not reach a significant level. The reason for this difference may be that regions hosting adjacent events are more susceptible to the positive radiation effects of large-scale events in neighboring provinces, achieving a bidirectional and efficient sports resource scheduling model. On the other hand, if two adjacent provinces host the event, it is difficult to coordinate the joint forces from different directions (ρ=−0.72), which limits the adjustment of sports resources. In summary, the “one neighbor” event hosting model has higher efficiency in the layout of sports resources.

## 6 Policy recommendations

1. It is recommended to build a policy concept of high-quality development, shifting the driving force of sports resources from pursuing scale and speed to pursuing efficiency, innovation, and sustainability. The high-quality growth of public sports activities should be an important indicator for identifying large-scale comprehensive sports events, implementing policy linkage, and optimizing factor growth mechanisms. From the perspective of organizational procedures, large-scale comprehensive sports events are decided and resource allocation is proposed by organizers. However, the local governments that host them usually focus more on their core responsibilities for the events, such as venue layout, process management, and personnel coordination, and do not pay much attention to the social efficiency of event resources, presenting a characteristic of “emphasizing competition and neglecting people's livelihood.” It is suggested to combine the current development status of regional public sports activities, systematically promoting planning layout, facility construction, service supply, and institutional mechanism innovation. Based on the implementation of the “Implementation Plan for the National Fitness Public Service System,” a diversified collaborative layout of “government led + market participation + social organization co construction” should be constructed to build an inclusive, intelligent, and sustainable high-quality event hosting model.
2. The layout of sports resources must be optimized and balanced and long-term regional development achieved. Large-scale comprehensive sports events can significantly increase the growth rate of local public sports activities for 3 years and have a spatial spillover effect. Taking the research sample as an example, among the 10 provinces that host the event, eight are located in the east and northeast regions, and only Shaanxi and Xinjiang are located in the west, while many provinces in the central region have not held them. Therefore, it is recommended that policies guide the central and western provinces to give priority to holding or jointly organizing large-scale comprehensive sports events to improve the backward status of public resources for sports services. On the other hand, large-scale comprehensive sports events can bring faster development of public sports activities to areas with more backward initial sports policy environments. They should focus on areas with weak sports policies and make adjustments, such as taking into account the needs of national fitness and competition in Henan, Anhui, and other populous provinces and building grassroots carriers such as “people's gyms” and “scientific fitness guidance centers” to lay a mass foundation for large-scale sports events.
3. There should be more focus on regional areas and the spatial layouts of intraprovincial and interprovincial events to plan the event structure, and regional coordination and industrial linkage in the sports service industry must be effectively improved. Based on the research results, the frequency of large-scale comprehensive sports events should be strictly controlled. Taking a 15-year research cycle as an example, holding a single event is the most ideal to drive the growth rate of regional public sports activities, which can bring 10% growth, while holding two similar events will significantly reduce the growth rate. Caution must be exercised to hold multiple large-scale events in different cities in the same province, because such events will significantly restrict the growth rate of regional public sports activities. The layout of interprovincial events gives priority to the spatial layout of two or two adjacent areas, whether it is too scattered (no contiguous provinces) or concentrated (more than two contiguous provinces), which will reduce the radiation efficiency of the growth of public sports activities.

## 7 Conclusion

Based on panel data from 31 provincial-level regions in China from 2008 to 2022, this study used the SDID method to estimate the driving mechanism of large-scale comprehensive sports events on public sports activities. The following conclusions were drawn: (1) During the 15-year period, the overall growth of public sports activities in various provinces in China showed a spatial clustering trend, and the “high-high” clustering areas were mostly provinces that had hosted large-scale comprehensive events. (2) Holding large-scale comprehensive sports events can significantly promote the growth rate of public sports activities in various provinces and regions, with an average annual growth rate of approximately 8.67% and a duration of about 3 years. After that, the growth rate was no longer significant, but it always maintained a positive trend. (3) Large-scale comprehensive sports events had a radiating effect on the growth of public sports activities. At a distance of 500 km, the positive radiation effect reached a significant level. (4) Large-scale comprehensive sports events mainly achieved rapid growth of public sports activities through three types of regulatory factors, regional balance, policy environment, and factor growth, in order to improve the uneven development of the region. (5) Over a period of 15 years, a large-scale comprehensive sports event held in a provincial-level region had the greatest driving effect on the growth rate of public sports activities, at approximately 10%, while events held in different cities within the province will lead to a significant decrease in growth rate, with an average decrease rate of approximately 8.1%. From the perspective of interprovincial event layout, the “two provinces adjacent” event hosting model was most conducive to achieving rapid growth in regional public sports activities, with a growth rate of approximately 9.16%.

There are also some shortcomings in this research, such as the low dimensionality of the sample data. In order to reduce collinearity and ensure regression quality, only six control variables were selected. The public sports and fitness data used were mainly obtained from middle-aged and elderly sports activity groups, making it difficult to cover ideal sports population panel data for all age groups. In the later period of the study period, the COVID-19 pandemic occurred in China, which led to a significant reduction in public outdoor sports activities after 2020, causing a large data fluctuation. Although indoor sports and fitness growth rates could be found as substitutes, there was a lack of unified and authoritative data publishing institutions. In addition, due to the scarcity of large-scale comprehensive sports events, there were no provinces in the central region to host such events, and therefore, the study of spatial agglomeration could not be extended to higher adjacent dimensions, also leading to certain limitations in the analysis results. Overall, this study reflects the rapid growth and short duration of public participation in sports activities, which requires further analysis and resolution from the perspectives of in-depth policy elements, supply side structural reform, and mechanism innovation.

## Data Availability

The original contributions presented in the study are included in the article/Supplementary Material, further inquiries can be directed to the corresponding authors.
