# Supplementary material for: The driving mechanism of large-scale comprehensive sports events on regional public sports activity growth: evidence from China's provincial panel data
Source: Front Sports Act Living. 2026 Aug 10;8:1883093. doi: 10.3389/fspor.2026.1883093 (PMC13500525; doi:10.3389/fspor.2026.1883093)
Supplement: Supplementary file 1 [file Table1.docx]

**Table1. Descriptive statistics and panel structure.**

**Descriptive Statistics**

| **Variable** | **Obs** | **Mean** | **Std. dev.** | **Min** | **Max** |
| --- | --- | --- | --- | --- | --- |
| DV | 465 | 5.269258 | 12.75677 | -18.049 | 49.827 |
| MSE | 465 | 7.993588 | .9172963 | 6.479121 | 14.03278 |
| ISV | 465 | 9.307082 | 1.216738 | 2 | 11.85974 |
| NSCO | 465 | 4.575973 | .8934699 | 3.453932 | 8.579989 |
| NSF | 465 | 5.775069 | 1.756314 | 0 | 9.69289 |
| IDE | 465 | 7.512866 | 1.727427 | 3.2 | 10.64913 |
| ISFC | 465 | 9.78063 | 1.496693 | 3.735763 | 12.89498 |

DV=dependent variable; MSE=mass sports expenditure; ISV=investment in sports venues; NSCO= number of sports club organizations; NSF=number of sports fields built and maintained; IDE=investment in domestic events; ISFC=Investment in sports facility construction

**Panel Structure**

| Variable | 1 | Mean | Std. dev. | Min | Max | Observations |
| --- | --- | --- | --- | --- | --- | --- |
| DV | overall | 5.269258 | 12.75677 | -18.049 | 49.827 | N=465 |
|  | between |  | 2.836063 | .0888666 | 11.71347 | n=31 |
|  | within |  | 12.44727 | -17.8499 | 50.94386 | T=15 |
| MSE | overall | 7.993588 | .9172963 | 6.479121 | 14.03278 | N=465 |
|  | between |  | .7443376 | 6.853978 | 9.730919 | n=31 |
|  | within |  | .5514624 | 6.294546 | 12.29545 | T=15 |
| ISV | overall | 9.30601 | 1.215646 | 2 | 11.85974 | N=465 |
|  | between |  | .8629889 | 7.044666 | 10.82765 | n=31 |
|  | within |  | .8692048 | 4.010148 | 12.02326 | T=15 |
| NSCO | overall | 4.575973 | .8934699 | 3.453932 | 8.579989 | N=465 |
|  | between |  | .7820098 | 3.765342 | 7.684851 | n=31 |
|  | within |  | .4529909 | 1.527335 | 8.30111 | T=15 |
| NSF | overall | 5.750691 | 1.756314 | 0 | 9.69289 | N=465 |
|  | between |  | .8859381 | 3.497635 | 7.428953 | n=31 |
|  | within |  | 1.524282 | 1.075948 | 11.41871 | T =15 |
| IDE | overall | 7.512866 | 1.727427 | 3.2 | 10.64913 | N=465 |
|  | between |  | .8522316 | 6.062452 | 9.124983 | n=31 |
|  | within |  | 1.509841 | 1.960143 | 10.80984 | T =15 |
| ISFC | overall | 9.78063 | 1.496693 | 3.735763 | 12.89498 | N=465 |
|  | between |  | .6962583 | 8.397729 | 11.16006 | n=31 |
|  | within |  | 1.330391 | 3.526055 | 12.69841 | T=15 |

DV=dependent variable; MSE=mass sports expenditure; ISV=investment in sports venues; NSCO= number of sports club organizations; NSF=number of sports fields built and maintained; IDE=investment in domestic events; ISFC=Investment in sports facility construction.
