# Supplementary material for: The driving mechanism of large-scale comprehensive sports events on regional public sports activity growth: evidence from China's provincial panel data
Source: Front Sports Act Living. 2026 Aug 10;8:1883093. doi: 10.3389/fspor.2026.1883093 (PMC13500525; doi:10.3389/fspor.2026.1883093)
Supplement: Supplementary file 2 [file Table2.docx]

**Table2. Regression results of figure 2.**

Fixed-effects (within) regression	Number of obs	=	465

Group variable: prov	Number of groups =	31

R-squared: Obs per group:

Within = 0.1762	min =	15

Between = 0.0311	avg =	15.0

Overall = 0.1599	max =	15

F(21,30)	=	30.29

corr(u_i, Xb) = -0.1394	Prob > F	=	0.0000

(Std. err. adjusted for 31 clusters in prov)

------------------------------------------------------------------------------

| Robust

psag | Coefficient std. err. t	P>|t|	[95% conf. interval]

-------------+----------------------------------------------------------------

dm0 | -.1902122	5.659986	-0.03	0.973	-11.74945	11.36902

dm1 |	6.239641	3.286189	1.90	0.067	-.4716518	12.95093

dm2 | -.9150407	3.244846	-0.28	0.780	-7.541901	5.711819

dm3 | -1.290779	3.467237	-0.37	0.712	-8.371822	5.790264

dm4 |	1.404883	2.579883	0.54	0.590	-3.863941	6.673706

dm5 |	2.347616	2.995225	0.78	0.439	-3.769449	8.464682

dm6 |	1.606588	3.162742	0.51	0.615	-4.852593	8.065769

|

year |

2009 | -7.963085	3.625472	-2.20	0.036	-15.36729	-.5588836

2010 | -2.530061	4.170534	-0.61	0.549	-11.04743	5.987305

2011 | -3.386158	4.506752	-0.75	0.458	-12.59017	5.817858

2012 | -7.185045	3.254852	-2.21	0.035	-13.83234	-.5377501

2013 |	9.723846	4.518625	2.15	0.040	.4955838	18.95211

2014 | -.1634301	3.916658	-0.04	0.967	-8.162312	7.835452

2015 | -8.083817	3.953286	-2.04	0.050	-16.1575	-.010131

2016 | -3.850978	3.835029	-1.00	0.323	-11.68315	3.981196

2017 | -7.722753	3.458523	-2.23	0.033	-14.786	-.6595056

2018 |	-5.31443	4.357642	-1.22	0.232	-14.21392	3.585062

2019 | -5.357107	3.817618	-1.40	0.171	-13.15372	2.439509

2020 | -2.991075	4.322514	-0.69	0.494	-11.81883	5.836675

2021 |	.3367951	3.882552	0.09	0.931	-7.592434	8.266024

2022 |	5.622698	4.695441	1.20	0.240	-3.966672	15.21207

|

_cons |	6.834841	3.116731	2.19	0.036	.4696274	13.20006

-------------+----------------------------------------------------------------

sigma_u |	3.08999

sigma_e | 11.975128

rho | .06242518	(fraction of variance due to u_i)

------------------------------------------------------------------------------

DV=dependent variable; MSE=mass sports expenditure; ISV=investment in sports venues; NSCO= number of sports club organizations; NSF=number of sports fields built and maintained; IDE=investment in domestic events; ISFC=Investment in sports facility construction.
