# Supplementary material for: The driving mechanism of large-scale comprehensive sports events on regional public sports activity growth: evidence from China's provincial panel data
Source: Front Sports Act Living. 2026 Aug 10;8:1883093. doi: 10.3389/fspor.2026.1883093 (PMC13500525; doi:10.3389/fspor.2026.1883093)
Supplement: Supplementary file 3 [file Table3.docx]

**Table 3. Regression results of figure 3.**

Fixed-effects (within) regression	Number of obs	=	465

Group variable: prov	Number of groups =	31

R-squared: Obs per group:

Within = 0.2724	min =	15

Between = 0.0669	avg =	15.0

Overall = 0.2468	max =	15

F(29,30)	=	.

corr(u_i, Xb) = -0.1280	Prob > F	=	.

(Std. err. adjusted for 31 clusters in prov)

------------------------------------------------------------------------------

| Robust

psag | Coefficient std. err. t	P>|t|	[95% conf. interval]

-------------+----------------------------------------------------------------

pre7 |	-2.67528	1.886659	-1.42	0.166	-6.528352	1.177792

pre6 | -.7073326	3.832302	-0.18	0.855	-8.533936	7.119271

pre5 |	1.907164	3.331719	0.57	0.571	-4.897115	8.711442

pre4 | -.6192563	2.654054	-0.23	0.817	-6.039557	4.801044

pre3 | -1.532176	2.238009	-0.68	0.499	-6.102799	3.038448

pre2 |	.9965397	4.607239	0.22	0.830	-8.412699	10.40578

pre1 | -.9457324	2.93844	-0.32	0.750	-6.946826	5.055362

current |	2.727232	3.303234	0.83	0.416	-4.018873	9.473336

post1 |	6.354503	2.62786	2.42	0.022	.9876962	11.72131

post2 |	10.66998	1.702763	6.27	0.000	7.192471	14.14748

post3 |	6.940435	2.051133	3.38	0.002	2.751463	11.12941

post4 |	1.717893	2.357753	0.73	0.472	-3.097281	6.533068

post5 |	3.754577	2.510423	1.50	0.145	-1.37239	8.881544

post6 |	3.063772	1.866638	1.64	0.111	-.7484122	6.875955

post7 | -.2564411	1.792175	-0.14	0.887	-3.916552	3.403669

lnMSE |	2.214505	1.068764	2.07	0.047	.0317971	4.397212

lnISV | -.4480921	.7494971	-0.60	0.554	-1.978769	1.082585

lnNSCO | -.2884339	.8041817	-0.36	0.722	-1.930792	1.353924

lnNSF |	.3168198	.5578087	0.57	0.574	-.8223776	1.456017

lnIDE | -.6088259	.3760515	-1.62	0.116	-1.376826	.1591738

lnISFC |	.1286583	.5141834	0.25	0.804	-.9214443	1.178761

|

year |

2009 | -.9228528	1.336994	-0.69	0.495	-3.653358	1.807652

2010 |	.9795569	1.841723	0.53	0.599	-2.781743	4.740856

2011 |	2.419349	2.518173	0.96	0.344	-2.723446	7.562144

2012 |	.8614138	2.636552	0.33	0.746	-4.523145	6.245972

2013 |	17.26336	4.369882	3.95	0.000	8.338873	26.18785

2014 |	7.70122	3.823886	2.01	0.053	-.1081976	15.51064

2015 |	1.064764	3.51668	0.30	0.764	-6.117255	8.246783

2016 |	6.677802	4.376823	1.53	0.138	-2.260863	15.61647

2017 |	4.578485	3.921257	1.17	0.252	-3.429789	12.58676

2018 |	6.979235	4.108943	1.70	0.100	-1.412347	15.37082

2019 |	6.767201	4.451673	1.52	0.139	-2.324328	15.85873

2020 |	9.761167	4.356262	2.24	0.033	.8644936	18.65784

2021 |	10.12733	3.800669	2.66	0.012	2.365328	17.88933

2022 |	18.10048	5.077682	3.56	0.001	7.730469	28.47049

|

_cons | -17.78196	18.42894	-0.96	0.342	-55.41887	19.85495

-------------+----------------------------------------------------------------

sigma_u | 3.0938764

sigma_e | 9.5107515

------------------------------------------------------------------------------

DV=dependent variable; MSE=mass sports expenditure; ISV=investment in sports venues; NSCO= number of sports club organizations; NSF=number of sports fields built and maintained; IDE=investment in domestic events; ISFC=Investment in sports facility construction.
