# Supplementary material for: The driving mechanism of large-scale comprehensive sports events on regional public sports activity growth: evidence from China's provincial panel data
Source: Front Sports Act Living. 2026 Aug 10;8:1883093. doi: 10.3389/fspor.2026.1883093 (PMC13500525; doi:10.3389/fspor.2026.1883093)
Supplement: Supplementary file 4 [file Table4.docx]

**Table4. Placebo test results.**

**Time-placebo test with a 5-period delay**

------------------------------------------------------------------------------

Coefficient	Std. err. z	P> z	[95% conf. interval]

-------------+----------------------------------------------------------------

Tdid

1. 2.027302	2.734176	0.74	0.458	-3.331584	7.386188

2. 1.762687	1.664702	1.06	0.290	-1.50007	5.025444

3. 1.266643	1.918193	0.66	0.509	-2.492946	5.026233

4. 2.454841	1.718929	1.43	0.153	-.9141977	5.82388

5. 5.268623	1.707479	3.09	0.002	1.922025	8.615221

------------------------------------------------------------------------------

**Spatial placebo test**

--------------------------------------------------------------

P-value

Coefficient	Two-sided	left-sided	right-sided

-----------+-------------+------------------------------------

Tdid	6.705823	0.0200	0.9960	0.0040

--------------------------------------------------------------

**Tdid, time-varying difference-in-difference.**
