# Supplementary material for: The driving mechanism of large-scale comprehensive sports events on regional public sports activity growth: evidence from China's provincial panel data
Source: Front Sports Act Living. 2026 Aug 10;8:1883093. doi: 10.3389/fspor.2026.1883093 (PMC13500525; doi:10.3389/fspor.2026.1883093)
Supplement: Supplementary file 5 [file Table5.docx]

**Table 5. Statistics of three types of competitions (Held from 2008 to 2022)**

| **Annual** | **Professional leagues and tournaments D1** | **Special events t D2** | **General international competitions D3** |
| --- | --- | --- | --- |
| 2009 | ‌ WMA Chinese Martial Arts Professional League Anhui | National International Wrestling Championship, Jiangsu | Sudirman Cup Badminton Championships, Guangzhou |
|  | National Women's Volleyball Championship in Jiangsu | China Tennis Open in Beijing | World Snooker Grand Prix in Guangzhou |
| 2010 | China Intercontinental Club Championship Beijing | Sudirman Cup Badminton Mixed Team Competition Qingdao Shandong | ‌Snooker China Open Beijing |
|  |  |  | Asian Para Games Guangzhou |
| 2011 | Modern Pentathlon Championships in Guangzhou |  | Swimming World Championships in Shanghai |
|  |  |  | Shandong for the Asian Beach Games |
| 2012 | ‌ Chinese Football Association Super League (CSL) Guiyang | National Race Walking Championships Huai'an |  |
|  | Chinese FA Cup Guangzhou | Hangzhou International Marathon |  |
|  |  | Zhengzhou-Kaifeng International Marathon |  |
| 2013 | Guangzhou Evergrande AFC Champions League Guangzhou | National Student Games Women's Volleyball Competition Shanghai | ‌ Table Tennis Team World Cup 2013 Guangzhou |
| 2014 | CBA All-Star Game Beijing |  | Asian Basketball Championship Wuhan, Hubei |
| 2015 |  | National Women's Volleyball Championship, Fujian | Beijing, World Athletics Championships |
|  |  | National Cross-Country Championships in Yunnan | Suzhou World Table Tennis Championships |
|  |  |  | Asian Athletics Championships, Wuhan, Hubei |
| 2016 |  | "I Love Football" China Football Folk Championship in Hunan | World Judo Grand Prix in Shandong |
|  |  | Yunnan National Martial Arts Routine Championship |  |
|  |  | Wuhan International Marathon |  |
| 2017 | ‌ National Women's Volleyball Championship, Taicang, Jiangsu | Xi'an International Marathon | World Roller Skating Championships in Nanjing |
|  |  | Hengshui International Marathon |  |
|  |  |  |  |
| 2018 | National Athletics Championships, Taiyuan, Shanxi |  | ‌ IAAF Diamond League (Shanghai stop) |
|  | ‌ Guangdong in the National Women's Volleyball Championship |  | World Badminton Championships, Nanjing, Jiangsu |
| 2019 |  | National Weightlifting World Cup in Fujian, | ‌ World Military Games in Wuhan |
|  |  |  | Sudirman Cup World Badminton Mixed Team Championships, Nanning, Guangxi |
|  |  |  | World Wushu Championships in Shanghai |
| 2020 | CBA Resumed Season, Guangdong |  |  |
|  | Henan National Men's Boxing Championships |  |  |
| 2021 | National Athletics Championships in Chongqing |  |  |
|  | Hebei National Men's Volleyball Championship |  |  |
| 2022 | National Table Tennis Championships, Huangshi, Hubei; |  | Chengdu Universiade 2022 |
|  |  |  | ‌ IAAF Diamond League Shanghai |
|  |  |  | Yangzhou World Half Marathon Championships |
