# Supplementary material for: The driving mechanism of large-scale comprehensive sports events on regional public sports activity growth: evidence from China's provincial panel data
Source: Front Sports Act Living. 2026 Aug 10;8:1883093. doi: 10.3389/fspor.2026.1883093 (PMC13500525; doi:10.3389/fspor.2026.1883093)
Supplement: Supplementary file 6 [file Table6.docx]

**Table6. Excluding the provinces hosting events in the first two cycles of 2008.**

Excluding the provinces that held large-scale comprehensive sports events from 2001 to 2007.

| **Year** | **City** | **Province** | **Events** |
| --- | --- | --- | --- |
| 2001 | Guangzhou | Guangdong Province | National Games |
| 2001 | Beijing | Beijing | University Games |
| 2003 | Harbin | Heilongjiang Province | Harbin Winter Sports Meeting |
| 2005 | Nanjing | Jiangsu Province | National Games |
| 2007 | Changchun | Jilin Province | Changchun Asian Games |
