# Supplementary material for: The driving mechanism of large-scale comprehensive sports events on regional public sports activity growth: evidence from China's provincial panel data
Source: Front Sports Act Living. 2026 Aug 10;8:1883093. doi: 10.3389/fspor.2026.1883093 (PMC13500525; doi:10.3389/fspor.2026.1883093)
Supplement: Supplementary file 7 [file Table7.docx]

**Table 7. DEA model estimation results**

**Min-Max Normalization**

Mapping the data range to the interval of [0,1], the specific method is:

Where max (x) is the maximum value of the variable, and min (x) is the minimum value of the variable.

**CCR method**

The CCR model calculates the resource allocation efficiency under the condition of constant return to scale. Taking the efficiency index of the j_0_th decision unit as the objective and the efficiency of all decision units as the constraint, the following model is adopted:

Among them, x_ij_ represents the total amount of investment in the i-th input element by the jth decision unit, y_rj_ represents the total output of the r-th product in the jth decision unit, and v_i_ and u_r_ respectively refer to the weight coefficients of the i-th type of input and the r-th type of output.

**Policy evaluation: Dmu（Decision-making unit） scores for 31 provinces**

| **DMU Score for investment in competitive sports** | | | | **DMU Score for investment in mass sports** | | | |
| --- | --- | --- | --- | --- | --- | --- | --- |
| dmu:1 | 29 | .172488 | . | dmu:1 | 47 | .0545234 |  |
| dmu:2 | 58 | .0239802 |  | dmu:2 | 26 | .215521 |  |
| dmu:3 | 62 | .0104625 |  | dmu:3 | 44 | .0849192 |  |
| dmu:4 | 34 | .110519 |  | dmu:4 | 27 | .207676 |  |
| dmu:5 | 7 | .561947 |  | dmu:5 | 25 | .218494 |  |
| dmu:6 | 31 | .144225 |  | dmu:6 | 40 | .0944701 |  |
| dmu:7 | 46 | .0564756 |  | dmu:7 | 21 | .244283 |  |
| dmu:8 | 53 | .0368763 |  | dmu:8 | 59 | .0188039 |  |
| dmu:9 | 51 | .0371193 |  | dmu:9 | 10 | .46092 |  |
| dmu:10 | 19 | .262614 | . | dmu:10 | 14 | .371624 | . |
| dmu:11 | 41 | .091156 | . | dmu:11 | 23 | .22746 | . |
| dmu:12 | 50 | .0379616 | . | dmu:12 | 39 | .0947414 | . |
| dmu:13 | 36 | .10503 | . | dmu:13 | 60 | .0153996 | . |
| dmu:14 | 49 | .0405754 | . | dmu:14 | 9 | .46415 | . |
| dmu:15 | 55 | .0326998 | . | dmu:15 | 11 | .418463 | . |
| dmu:16 | 1 | 1 | . | dmu:16 | 42 | .0899313 | . |
| dmu:17 | 20 | .254066 | . | dmu:17 | 30 | .152985 | . |
| dmu:18 | 15 | .352799 | . | dmu:18 | 13 | .384041 | . |
| dmu:19 | 6 | .606072 | . | dmu:19 | 28 | .194273 | . |
| dmu:20 | 12 | .418436 | . | dmu:20 | 1 | 1 | . |
| dmu:21 | 57 | .0267038 | . | dmu:21 | 1 | 1 | . |
| dmu:22 | 22 | .237276 | . | dmu:22 | 8 | .561145 | . |
| dmu:23 | 43 | .0878968 | . | dmu:23 | 35 | .109251 | . |
| dmu:24 | 24 | .223949 | . | dmu:24 | 38 | .0962437 | . |
| dmu:25 | 32 | .134308 | . | dmu:25 | 45 | .0585327 | . |
| dmu:26 | 48 | .0441874 | . | dmu:26 | 33 | .11577 | . |
| dmu:27 | 54 | .0352156 | . | dmu:27 | 37 | .0989327 | . |
| dmu:28 | 1 | 1 | . | dmu:28 | 5 | .629948 | . |
| dmu:29 | 61 | .0149634 | . | dmu:29 | 16 | .34289 | . |
| dmu:30 | 18 | .277544 | . | dmu:30 | 17 | .292479 | . |
| dmu:31 | 52 | .0369907 | . | dmu:31 | 56 | .0269713 | . |
